# α_1_-antitrypsin modulates microglial-mediated neuroinflammation and protects microglial cells from amyloid-β-induced toxicity

**DOI:** 10.1186/s12974-014-0165-8

**Published:** 2014-09-23

**Authors:** Maike Gold, Amalia M Dolga, Janine Koepke, David Mengel, Carsten Culmsee, Richard Dodel, Andreas Rembert Koczulla, Jan-Philipp Bach

**Affiliations:** Department of Neurology, University of Marburg, Baldingerstr, 35043 Marburg, Germany; Department of Pharmacology und Clinical Pharmacy, University of Marburg, Karl-v.-Frisch-Str. 1, 35043 Marburg, Germany; Department of Medicine, Pulmonary and Critical Care Medicine, University of Marburg, Baldingerstr, 35043 Marburg, Germany

**Keywords:** Calpain, Alpha-1 antitrypsin, Cytokine, Amyloid-β, Lipopolysaccharide (LPS), Alzheimer disease

## Abstract

**Background:**

One hallmark of Alzheimer disease is microglial activation. Therapeutic approaches for this neurodegenerative disease include the modulation of microglial cells. α_1_-antitrypsin (A1AT) has been shown to exert anti-inflammatory effects on macrophages and lung epithelial cells and an inhibition of calpain activity in neutrophil granulocytes. Nothing is known about the effect of A1AT on microglial-mediated neuroinflammation. Our aim was to investigate the effect of A1AT on amyloid-β (Aβ)- and LPS-treated microglial cells *in vitro* with respect to cytokine production, stress pathways, cell viability, phagocytotic abilities and the underlying mechanisms.

**Methods:**

Primary microglial cells were isolated from Swiss Webster mouse embryos on embryonic day 13.5. Cytokines in the supernatants of treated primary microglial cells were analyzed with ELISAs, and accumulated nitrite was detected with Griess reagents. Intracellular stress pathways were investigated in cell lysates using western blotting. Intracellular calcium levels were detected in BV-2 microglial cells loaded with the Ca^2+^-sensitive (fluorescent) dye Fluo-4. Calpain activity in primary microglial cells was assessed by using a calpain activity assay. Cell viability of Aβ-treated microglial cells was analyzed using MTT assay. Phagocytosis of Aβ was evaluated with western blot analysis.

**Results:**

Upon co-administration, A1AT reduced pro-inflammatory mediators induced by LPS or Aβ. Interestingly, we detected a reduction in calpain activity and in the concentration of intracellular calcium that might mediate the anti-inflammatory effects of A1AT. Inhibition of the classic activation pathways, such as phosphorylation of mitogen-activated protein kinases or activation of protein kinase A were excluded as a mechanism of A1AT-mediated effects. In addition, A1AT increased the viability of Aβ-treated microglial cells and reduced Aβ phagocytosis.

**Conclusions:**

We provide evidence on the mechanism of action of A1AT on microglial-mediated neuroinflammation *in vitro*. Our *in vitro* data indicate that A1AT treatment modulates microglial cells in inflammatory conditions and that this modulation is due to an inhibition of calpain activity and intracellular calcium levels. The underlying mechanisms of the effects observed here are promising for future therapeutic strategies and should thus be further pursued in transgenic mouse models of Alzheimer disease.

## Background

Alzheimer disease (AD) is the most common neurodegenerative disease leading to the loss of cholinergic neurons and their associated neuronal connections in the human brain. Pathological hallmarks are the extracellular formation of amyloid-β (Aβ) plaques and the intracellular formation of neurofibrillary tangles, composed of hyperphosphorylated tau-protein [1]. Besides its toxicity to neurons, Aβ activates microglial cells via different receptors expressed on the cell surface, such as toll-like receptors (TLR) [2]. Intracellular receptor signalling involves the classical mitogen-activated protein kinase (MAPK) pathways resulting in the nuclear translocation of nuclear factor κB (NFκB) [3] but also activation of cAMP/protein kinase A (PKA)/phosphorylated cAMP response element binding protein (CREB) [4]. Upon activation, microglial cells express and secrete pro-inflammatory factors, thereby generating an even more neurotoxic milieu [5]. The concentration of intracellular calcium also seems to play a role in the control of pro-inflammatory mediator release [6], and modulation of intracellular calcium levels can reduce the secretion of pro-inflammatory factors [7]. On the other hand, microglial cells also show neuroprotective effects through the uptake and degradation of toxic Aβ oligomers [8].

One therapeutic approach for AD is to modify neuroinflammation towards an activation state that is characterized by reduced production of pro-inflammatory mediators and increased Aβ clearance [9,10]. Recently, a new anti-inflammatory role for α_1_-antitrypsin (A1AT) has been described for activated monocytes as well as for lung endothelial cells [11,12]. The serine protease inhibitory activity mediated by A1AT protein is well acknowledged for its essential role in the degradation of neutrophil elastase in the lung [13]. A1AT proteins are also known for their inflammatory properties as acute phase proteins being produced in large amounts under inflammatory conditions [14]. Patients suffering from inborn A1AT deficiency are predisposed to lung diseases and to liver cirrhosis, mainly attributed to an accumulation of misfolded A1AT proteins [15]. As a therapy for inborn A1AT deficiency, substitution with pooled human A1AT is available. A1AT has also been shown to reduce calpain activity in neutrophil granulocytes [16]. Calpain, a key enzyme in the progress of neurodegenerative pathology, is activated by elevated intracellular calcium levels and it is strongly associated with endoplasmic reticulum stress and apoptosis [17]. In postmortem brains of AD patients, overactivation of calpain can be detected [18]. Recently, it was demonstrated that calpain activity is also involved in the activation of immune cells in a colitis mouse model [19].

In the present study, therefore, the effect of A1AT on microglial-mediated neuroinflammation and Aβ toxicity was investigated in murine primary microglial cells and the rodent microglial cell line BV-2.

## Methods

All chemicals were obtained from Sigma-Aldrich, St. Louis, MO, USA, unless otherwise indicated.

### Reagents and antibodies

*Prolastin* (Grifols, Barcelona, Spain, for more information see http://www.grifols-pi.info/inserts/Prolastin-C.pdf) was used as a source for A1AT. *Prolastin* (1 g) was solubilized with 40 ml ultrapure water, aliquoted and stored at -80°C until use.

To obtain Aβ oligomer-enriched preparations, 1 mg Aβ_1-42_ (Bachem, Bubendorf, Switzerland) was dissolved in 10% ammonia, aliquoted, freeze dried and stored at -80°C. Before use, Aβ_1-42_ aliquots were dissolved in sterile water (1 mg/ml), and then 100 mM Tris and 50 mM NaCl were added to obtain a 58 μM solution. A magnetic stirrer was added and the Aβ solution was stirred for 48 hours at 1,400 rpm at room temperature.

Antibodies against the following proteins were used: phosphorylated (phospho)-p38, phospho-p44/42, phospho-JNK (c-Jun N-terminal kinase)/SAPK (stress-activated protein kinase), phospho-CREB (all Cell Signaling, Danvers, MA, USA), Aβ (clone 6E10, Covance, Princeton, NJ, USA), A1AT, Vinculin, glyceraldehyde 3-phosphate dehydrogenase (GAPDH) (Novus Biologicals, Littleton, CO, USA) and horseradish peroxidase-conjugated secondary antibody goat anti-mouse, goat anti-rabbit (both Cell Signaling, Danvers, MA, USA) and donkey anti-goat (Santa Cruz Biotechnology, Dallas, TX, USA).

### Cells

Primary microglia cells were derived from Swiss Webster mouse embryos on embryonic day 13.5. All experimental protocols were approved by the office of the district president and the Institutional Animal Care and Use Committee of the University of Marburg. The study received institutional review board and experiments were carried out in accordance with EU Directive 1020/63/EU on the protection of animals used for scientific purposes. The procedure has been described in detail by Roettger *et al*. [20]. Briefly, mice were sacrificed, embryos were removed and mesencephalons were dissected and collected in Leibovitz L-15 media (PAA Laboratories GmbH, Pasching, Austria). Mesencephalons were homogenized by pipetting 30 times. Then, 5 ml Leibovitz L-15 medium was added and the solution was left for 10 minutes to remove cell debris. Next, 5 ml was removed into a new tube, centrifuged for 5 min at 300 g and the supernatant was then resuspended in Dulbecco’s modified Eagle’s medium (DMEM) supplemented with 10% fetal bovine serum (FBS), 1% streptomycin/penicillin and 1% L-glutamine (all PAA Laboratories GmbH, Pasching, Austria). Cells were seeded on polyethyleneimine-coated plates, cultivated at 37°C in a humidified atmosphere of 5% CO_2_, and after 7 days, 5 ng/ml granulocyte-macrophage colony-stimulating factor (Roche, Basel, Switzerland) was added to increase the yield of microglia. After 15 to 19 days in culture, cells were subplated and used for further experiments. For experiments, cell treatments were performed 24 hours after seeding. More than 95% of cells obtained were microglia as quantified by CD11b FACS analysis.

BV-2 cells (a kind gift of Jens Neumann, Magdeburg, Germany) were cultivated in DMEM supplemented with 10% FBS, 1% penicillin/streptomycin and 1% L-glutamine.

### Measurement of TNF-α, IL-6 and IL-1β

Before cell treatment, media was changed to serum-free media. Primary microglial cells were treated with 0.1 μg/ml LPS or 10 μM Aβ_1-42_ and 2 or 4 mg/ml A1AT. After 24 hours, the concentration of the pro-inflammatory cytokines TNF-α and IL-6 was measured in cell culture supernatants using the Duoset Enzyme-linked immunosorbent assay (ELISA) system (R&D, Minneapolis, MN, USA) according to the manufacturer’s protocol. Supernatants of cells treated with LPS and/or A1AT were also subjected to an IL-1β ELISA (R&D, Minneapolis, MN, USA). The absorbance was measured at 450 nm using a plate reader (ELISA-Reader Infinite™ 200series, Tecan, Crailsheim, Germany).

### Measurement of nitric oxide

Accumulated nitrite, a stable oxidation product of nitric oxide (NO), was measured using Griess reagents [21]. For this measurement, 50 μl of primary microglial cell supernatants was transferred to a 96-well microtiter plate, and 50 μl of solution 1 (1% sulfanilamide in 5% phosphoric acid) was added. After 10 minutes incubation in the dark, 50 μl of solution 2 (0.1% naphthylethylenediamine dihydrochloride) was added and incubated for 10 more minutes in the dark. The absorbance was measured at 550 nm using a plate reader (ELISA-Reader Infinite™ 200series, Tecan, Crailsheim, Germany).

### Cell lysis and western blot

Media was changed to serum-free media, and primary microglial cells were treated for the indicated times, washed twice with ice-cold phosphate-buffered saline and lysed for 5 minutes with M-Per lysis buffer (Thermo Scientific, Rockford, IL, USA) containing protease inhibitors and phosphatase inhibitors (Roche, Basel, Switzerland). Cells were harvested with a cell scraper and pelleted for 10 minutes at 14,000 rpm. Supernatants were transferred to a new tube, and protein concentration was determined using NanoDrop (Peqlab, Erlangen, Germany). Next, 10 μg of total protein was separated by 4 to 12% sodium dodecyl sulphate-polyacrylamide gel electrophoresis (SDS-PAGE) and transferred onto nitrocellulose membranes. Membranes were blocked for one hour at room temperature and incubated with the appropriate primary antibody solution for phospho-p38 (1:4,000), phospho p44/42 (1:4,000), phospho JNK (1:4,000), phospho CREB (1:500), A1AT (1:2,000), Aβ (1:2,000), GAPDH (1:5,000) or Vinculin (1:5,000) at 4°C over night. Primary antibody was detected using a species-specific secondary antibody. Membranes were incubated with SuperSignal^TM^ West Dura Extended Duration Substrate (Thermo Scientific, Rockford, IL, USA) and exposed to an autoradiographic film (Thermo Scientific, Rockford, IL, USA). Band intensity levels were measured by GS-800™ calibrated densitometer (Bio-Rad, Hercules, CA, USA).

### Intracellular calcium measurements with Fluo-4

To assess intracellular calcium levels 1.5 × 10^4^ BV-2 cells were seeded in a 96-well black plate, washed with Hank’s buffered salt solution (HBSS) and loaded with the calcium indicator Fluo-4 (5 μM) for 30 minutes at 37°C. The dye was removed, cells were kept in HBSS and baseline calcium levels for each well were assigned. The fluorescence of each well was determined with an automated FLUOstar Optima reader (BMG Labtechnologies GmbH, Offenburg, Germany) using an excitation wavelength of 485 nm and an emission wavelength of 520 nm.

To measure the effect of A1AT on intracellular calcium levels, we treated cells with 0.1 μg/ml LPS and/or 2 and 4 mg/ml A1AT and fluorescence was measured every 60 seconds for a time course of 200 minutes.

### Calpain activity assay

To measure the calpain activity of primary microglial cells, cells were seeded in a 24-well plate and media was changed to serum-free media. Cells were treated with 0.5, 2 and 4 mg/ml A1AT for 24 hours and subjected to a calpain activity assay (Abcam, Cambridge, UK). The assay was performed according to the manufacturer’s protocol.

### Cell viability assessment with MTT reduction assay

Cell viability of Aβ_1-42_ oligomer-treated primary microglial cells and A1AT co-treated cells was measured using a 3-(4,5-dimethylthiazol-2-yl)-2,5-diphenyltetrazolium bromide (MTT) reduction assay. Media was changed to serum-free media, and cells were treated with Aβ_1-42_ oligomers (2 μM) and A1AT (0.5, 2 and 4 mg/ml) for 24 hours. Culture medium was changed to fresh medium containing 0.5 mg/ml MTT and incubated for one hour at 37°C in a humidified atmosphere of 5% CO_2_. Medium was removed and cells were lysed with dimethyl sulfoxide (AppliChem, Darmstadt, Germany). The absorption was measured at 570 nm on a plate reader (ELISA-Reader Infinite™ 200series, Tecan, Crailsheim, Germany). Absorption values were normalized to untreated cells.

### Statistical analysis

All results are presented as the mean ± standard deviation of the mean. Differences between groups were evaluated for statistical significance using one-way analysis of variance (ANOVA) with Bonferroni *post-hoc* test. Data analysis was aided by XL Toolbox add-in for Excel, version 6.52 (xltoolbox.sourceforge.net).

For all statistical comparisons, the following definitions were used: (* = *P* value ≤ 0.05, ** = *P* value ≤ 0.01, *** = *P* value ≤ 0.001).

## Results

### α_1_-antitrypsin has an anti-inflammatory effect on lipopolysaccharide-treated primary microglial cells

**Figure 1:**
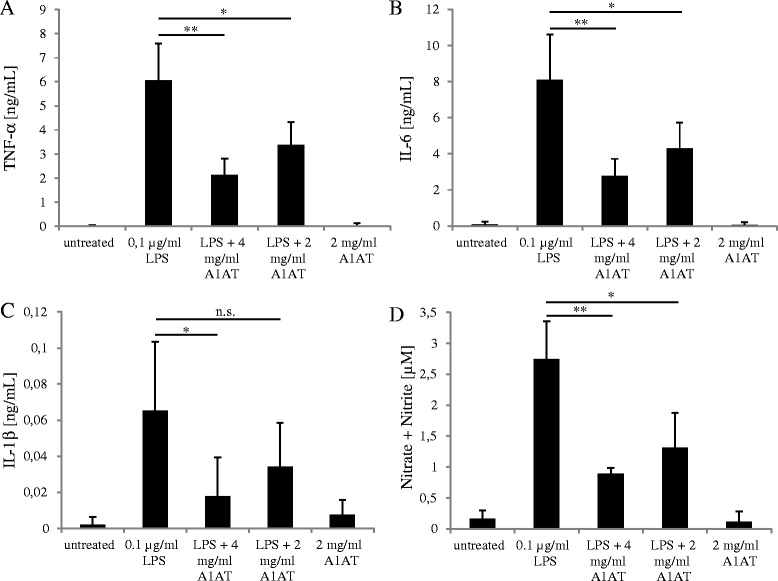
**α**_**1**_ **-antitrypsin (A1AT) reduces lipopolysaccharide (LPS)-induced release of pro-inflammatory mediators. A**-**C**. Cell culture supernatants of LPS and/or A1AT co-treated cells were subjected to ELISA to detect the levels of pro-inflammatory cytokines. Therefore, primary microglial cells were treated with 0.1 μg/ml LPS and/or 2 and 4 mg/ml A1AT for 24 hours, followed by quantification of TNF-α, IL-6, and IL-1β levels. Values are given in ng/ml. Means and standard deviations of the mean of three independent experiments are shown (*P value ≤ 0.05, **P value ≤ 0.01, n.s., not significant). **D**. Nitric oxide (NO) was measured in the same cell culture supernatants using Griess reagents. Values are given in μM. Means and standard deviations of the mean of three independent experiments are shown (*P value ≤ 0.05, **P value ≤ 0.01).

Primary microglial cells were treated for 24 hours with 0.1 μg/ml LPS and/or 2 and 4 mg/ml A1AT. Co-treatment with 4 mg/ml A1AT led to a two- to threefold reduction of TNF-α (*P* = 0.003), IL-6 (*P* = 0.002), IL-1β (*P =* 0.04) and NO levels (*P =* 0.006) in primary microglial cells. The lower dose of 2 mg/ml reduced TNF-α (*P =* 0.04), IL-6 (*P =* 0.02) and NO (*P =* 0.04) levels significantly (Figure 1A, B + D), whereas there was no effect on IL-1β (*P =* 0.16; Figure 1C). A1AT alone did not show any immunogenicity.

### Anti-inflammatory effect is not mediated via MAPK or PKA

**Figure 2:**
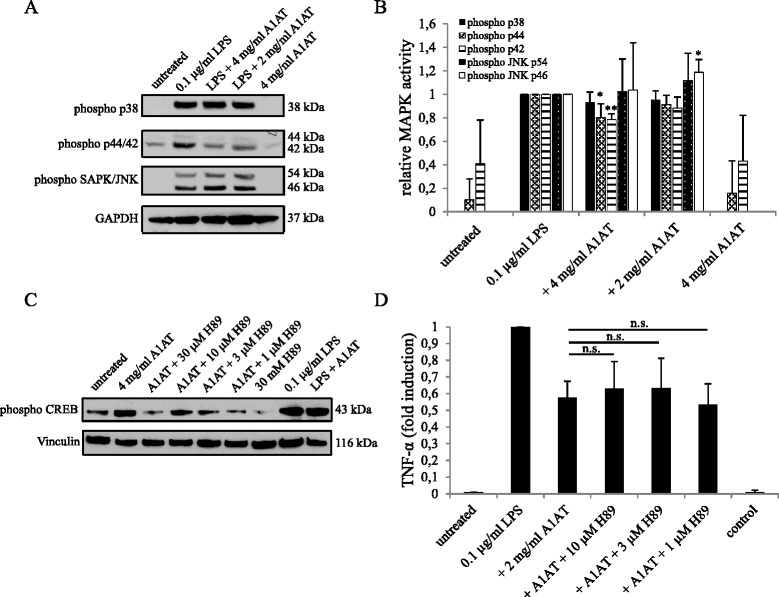
**α**_**1**_ **-antitrypsin (A1AT)’s reduction of pro-inflammatory mediators is independent of MAPK and PKA activation. A**. To detect the phosphorylation state of the MAPKs p38, p44/42 and JNK upon co-treatment with A1AT, primary microglial cells were incubated with 0.1 μg/ml lipopolysaccharide (LPS) and 2 and 4 mg/ml A1AT for 20 minutes, and cell lysates of the treated cells were subjected to western blotting. Antibodies against phospho-p38, phospho-p44/42 and phospho-JNK were used. GAPDH served as a loading control. One representative western blot out of three is shown. **B**. Band intensities of phosphorylated MAPK were measured by a densitometer and normalized with that of GAPDH. LPS-treated cells were set to 1. Means and standard deviations of the mean of three western blots are shown (*P value ≤ 0.05, **P value ≤ 0.01) **C**. To detect the effect of A1AT on the phosphorylation state of CREB, cells were pretreated with the PKA inhibitor H89 for 30 minutes and then treated with 4 mg/ml A1AT for 15 minutes or co-treated with 0.1 μg/ml LPS and 4 mg/ml A1AT for 15 minutes. Cell lysates were subjected to western blotting, and phospho-CREB was detected with a monoclonal antibody. Vinculin was used as a loading control. One representative western blot out of three is shown. **D**. To measure the impact on the release of the pro-inflammatory cytokine TNF-α, cells were pretreated with different concentrations of H89 and then treated with 0.1 μg/ml LPS and/or 2 mg/ml A1AT for 24 hours. TNF-α levels in cell culture supernatants of primary microglial cells were quantified with ELISA. Results were normalized to LPS-treated cells. Means and standard deviations of the mean of three independent experiments are shown.

It is well established that pro-inflammatory effects in immune cells are mediated via the phosphorylation of MAPK and transactivation of the transcription factor NF-κB. To investigate the correlation of A1AT with these classic inflammatory stress pathways, we analyzed the phosphorylation state of p38, p44/42 and SAPK/JNK upon treatment with 0.1 μg/ml LPS and A1AT after 20 minutes (Figure 2A). We saw a very slight negative correlation of 4 mg/ml A1AT on LPS-induced phosphorylation of p44/42 (*P =* 0.04/0.002) and a slight positive correlation in the phosphorylation state of JNK p46 with 2 mg/ml A1AT (*P =* 0.04); Figure 2B). However there was no change in the phosphorylation state of p38 with any A1AT co-treatment and no correlation with phospho p44/42 with 2 mg/ml A1AT co-treatment, a concentration that showed reductive effects on pro-inflammatory cytokines.

It has previously been reported that A1AT exerts its anti-inflammatory effect via activation of PKA [11], indicating an effect through the PKA/CREB pathway. Here, we detected increased phospho-CREB levels upon microglia treatment with 4 mg/ml A1AT and investigated whether this effect can be antagonized by different concentrations of the PKA-inhibitor H89 (1 to 30 μM). Indeed, we detected an antagonistic effect of H89 on the phosphorylation state of CREB (Figure 2C). However, the phosphorylation state of CREB was also increased in LPS and/or A1AT treated cells (Figure 2C). We further tested the effect of H89 on the anti-inflammatory effect of 2 mg/ml A1AT on LPS-treated cells. As shown in Figure 2D, the release of the pro-inflammatory cytokine TNF-α after LPS treatment could not be restored by different concentrations of H89 (1 to 10 μM). H89 treatment in the aforementioned concentrations had no effect on cytokine levels (data not shown).

### α_1_-antitrypsin reduces intracellular calcium-levels in BV-2 microglial cells

**Figure 3:**
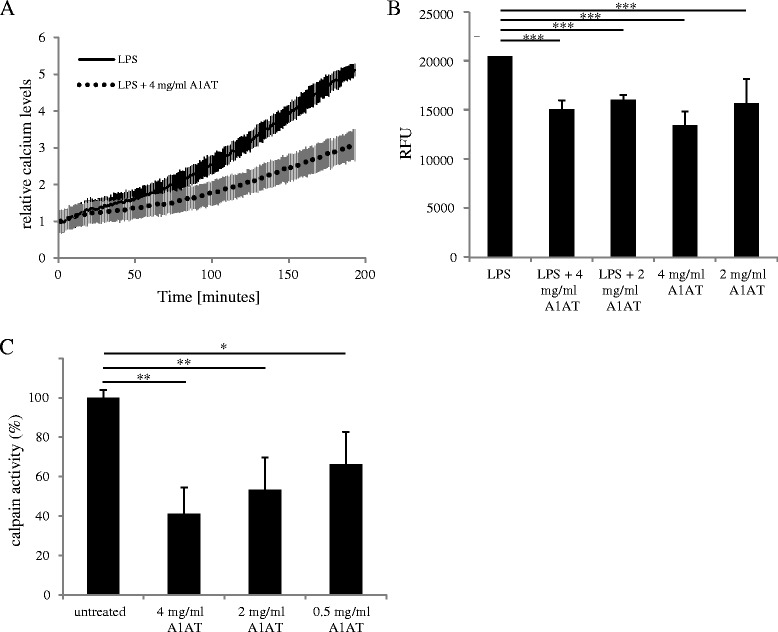
**α**_**1**_ **-antitrypsin (A1AT) reduces intracellular calcium levels and calpain activity in microglial cells.** To detect intracellular calcium levels, BV-2 microglial cells were loaded with Fluo-4 and baseline calcium levels for each well were measured and subtracted from the values. Cells were treated with 0.1 μg/ml lipopolysaccharide (LPS) and/or 2 and 4 mg/ml A1AT, and the course of intracellular calcium was measured over 200 minutes. RFU (relative fluorescence units) were normalized to time point zero. **A** shows the course of the values for LPS and LPS + 4 mg/ml A1AT co-treated cells. One representative experiment out of three is shown. **B** shows the mean values and standard deviations of the mean of the RFU of six wells after 100 minutes for LPS and/or A1AT-treated cells (***P value ≤ 0.001). **C**. Calpain activity was detected 24 hours after A1AT treatment with different concentrations of A1AT by using a calpain activity assay. Calpain activity of untreated cells was referred to as 100% activity. Means and standard deviations of the mean of three independent experiments are shown (*P value ≤ 0.05, **P value ≤ 0.01).

Calcium plays an important role in the activation of microglial cells, is required for calpain activation and is known to be enhanced in human AD brains [17,18]. Since we did not observe a strong regulative effect of A1AT on MAPK and the regulation of PKA does not seem to be responsible for the anti-inflammatory effect of A1AT, we investigated the change of intracellular calcium levels upon treatment with 0.1 μg/ml LPS and/or different concentrations of A1AT with the calcium fluorescent marker Fluo-4. For these experiments, we used BV-2 microglial cells because primary microglial cells change their shape dramatically after LPS treatment, which might lead to a misinterpretation of the results as Fluo-4 is a nonratiometric dye. Both concentrations applied here, 4 mg/ml A1AT as well as 2 mg/ml A1AT, reduced the rise of intracellular calcium over time by approximately 25% (LPS + 4 mg/ml: *P* = 0.0001, LPS + 2 mg/ml: *P* = 0.0002; Figure 3A, B). A1AT treatment alone also reduces intracellular calcium levels (4 mg/ml: *P* = 0.00004, 2 mg/ml: *P* = 0.02; Figure 3B).

### α_1_-antitrypsin reduces calpain activity in primary microglial cells

Al-Omari and colleagues showed an inhibitory role for A1AT on calpain activity in neutrophil granulocytes [16]. To investigate the effect of A1AT on calpain activity in primary microglial cells, we treated cells for 24 hours with 0.5, 2 and 4 mg/ml A1AT and subjected them to a calpain activity assay. A1AT reduced calpain activity in a dose-dependent manner (Figure 3C). By applying the highest concentration A1AT of 4 mg/ml, calpain activity could be reduced by 59% (*P* = 0.002). Also, 2 mg/ml A1AT reduced calpain activity by 47% (*P* = 0.002) and 0.5 mg/ml A1AT reduced calpain activity by 34% (*P* = 0.03). Treatment with bovine serum albumin using the same concentrations had no effect on calpain activity (data not shown).

### α_1_-antitrypsin has an anti-inflammatory effect on Aβ oligomer-treated primary microglial cells

**Figure 4:**
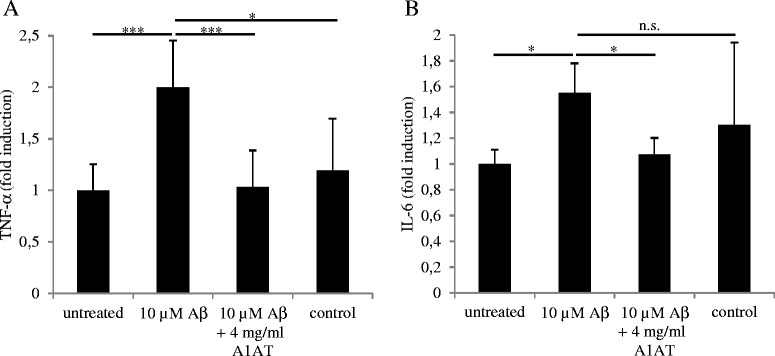
**α**_**1**_ **-antitrypsin (A1AT) reduces Aβ-induced release of TNF-α.** ELISA measurements were performed to detect the amount of the pro-inflammatory cytokines TNF-α **(A)** and IL-6 **(B)** in supernatants of Aβ_1-42_ oligomer and/or A1AT co-treated primary microglial cells 24 hours after treatment. Results were normalized to basal cytokine levels of untreated cells. Experiments were performed at least three times independently. Means and standard deviations of the mean of three independent experiments are shown (*P value ≤0.05, ***P value ≤0.001, n.s., not significant).

We were able to show an inhibitory effect of A1AT on cytokines in microglial cells upon LPS treatment. In AD it is assumed that Aβ oligomers induce the release of pro-inflammatory cytokines in microglial cells [2]. Here, we addressed the effect of A1AT on Aβ-induced inflammation in primary microglial cells. Therefore, cells were treated with 10 μM Aβ_1-42_ oligomers and co-treated with 4 mg/ml A1AT. The co-treatment with A1AT led to a significant reduction of TNF-α (*P* = 0.0008) and IL-6 (*P* = 0.03) levels (Figure 4A, B). The baseline concentration of untreated cells was 17 pg/ml for TNF-α and 12 pg/ml for IL-6.

### α_1_-antitrypsin treatment reduces Aβ oligomer-induced cytotoxicity in primary microglial cells

**Figure 5:**
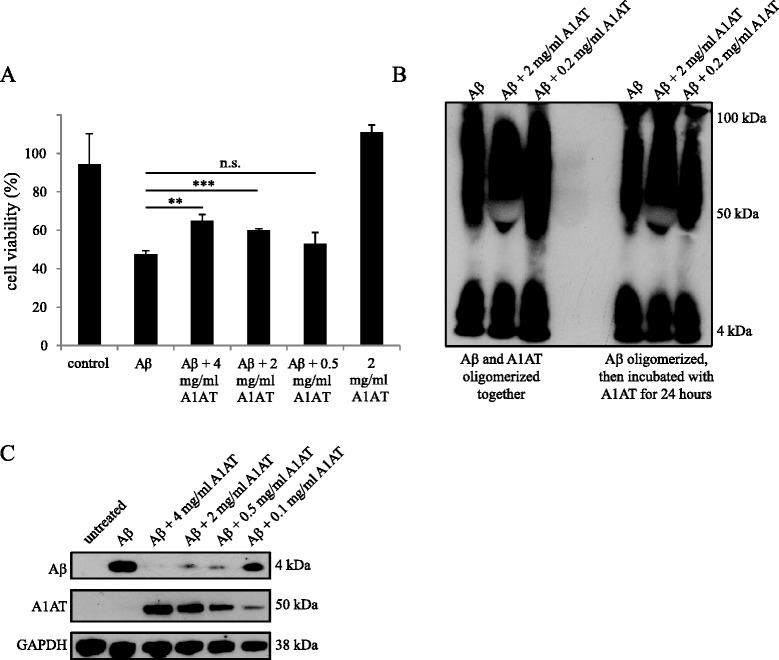
**α**_**1**_ **-antitrypsin (A1AT) treatment reduces Aβ oligomer-induced cytotoxicity on primary microglial cells. A**. Primary microglial cells were treated with 2 μM Aβ_1-42_ oligomers and/or different concentrations of A1AT for 24 hours. An MTT-assay was performed to detect the cell viability. Viability of untreated cells was referred to as 100% viability. Means and standard deviations of the mean of three independent experiments are shown (**P value ≤ 0.01, ***P value ≤ 0.001, n.s., not significant). **B**. 58 μM Aβ preparations were oligomerized and the effect of A1AT on the oligomerization process was assessed by western blotting with the monoclonal Aβ antibody 6E10. One representative western blot out of three is shown. **C**. Uptake of Aβ_1-42_ oligomers under the influence of different concentrations of A1AT was investigated by western blotting. Cells were treated with 2 μM Aβ and different concentrations of A1AT (4 mg/ml to 0.1 mg/ml) for three hours. We used the monoclonal antibody 6E10 to detect Aβ and a polyclonal antibody to detect A1AT in cell lysates. GAPDH was used as loading control. One representative western blot out of three is shown.

To detect the effect of A1AT on cell viability of Aβ_1-42_ oligomer-treated primary microglial cells, we treated cells with 2 μM Aβ_1-42_ oligomers and different concentrations of A1AT for 24 hours and subjected them to an MTT assay. Aβ_1-42_ oligomers reduced the viability to 48%. Co-treatment with different concentrations of A1AT exerted a positive effect on the cell viability in a dose-dependent manner (Figure 5A). With the highest concentration of A1AT, 4 mg/ml, cell viability was only reduced to 65% (*P =* 0.002), and with 2 mg/ml, cell viability was reduced to 60% (*P* = 0.0008). At 0.5 mg/ml A1AT, the lowest dose, the viability of the Aβ oligomer-treated cells did not improve (*P =* 0.28). To exclude unspecific protein effects, we used the same concentrations of bovine serum albumin, which had no effect on the cell viability of Aβ_1-42_-treated cells (data not shown).

### α_1_-antitrypsin has no impact on the oligomerization process of Aβ

It has been previously reported that α1-antichymotrypsin, a multifunctional glycoprotein of the serpin family [22], and A1AT show inhibitory effects on the formation of fibrils and associated toxicity using Aβ_25-35_ and physiological concentrations of α1-antichymotrypsin and A1AT [23]. To investigate the effect of A1AT on the oligomerization process of Aβ_1-42_, we performed western blot analysis using partly non-reducing conditions and detected Aβ with the monoclonal antibody 6E10. We further tested two different experimental setups. In the first experimental design we oligomerized Aβ_1-42_ in the presence of 0.2 and 2 mg/ml A1AT. In the second experimental design, we added 0.2 and 2 mg/ml A1AT after the oligomerization process and incubated the solution at 37°C for 24 hours with continuous agitation. Then, 2 μl of each solution was subjected to western blot analysis. No differences in the oligomerization process could be detected in any of the two conditions (Figure 5B). Thus, the benefit of A1AT proteins on Aβ_1-42_ toxicity is not attributed to an effect of A1AT on the aggregation process of Aβ_1-42_.

### α_1_-antitrypsin has an inhibitory effect on Aβ phagocytosis

The influence of A1AT on microglial phagocytosis of Aβ_1-42_ oligomers was assessed by western blot analysis. Therefore, primary microglial cells were treated with 2 μM Aβ_1-42_ oligomers and different concentrations of A1AT (0.1 to 4 mg/ml) for three hours. Cell lysates were subjected to western blot analysis and phagocytosed Aβ was detected with the monoclonal anti-Aβ antibody 6E10 (Figure 5C). Co-treatment with A1AT led to a strong inhibition of Aβ_1-42_ uptake, whereas different concentrations of Aβ_1-42_ had no effect on the uptake of A1AT (data not shown); thus we can exclude a competitive effect.

## Discussion

Compelling evidence suggests that activation of glia and overexpression of glial cytokines are early events in the pathogenesis of AD [24-26]. Toxic Aβ oligomers induce neuroinflammation [27] and also reduce the viability of microglial cells [28]. A1AT has been assigned an anti-inflammatory role in the activation of human monocytes as well as of lung epithelial cells [11,12]. We therefore were interested in the effect of A1AT in microglial-mediated neuroinflammation and investigated the effect of A1AT on Aβ and LPS-induced cytokines, intracellular calcium levels, calpain activity, Aβ toxicity, Aβ aggregation and Aβ phagocytosis in primary microglial or BV-2 microglial cells.

Indeed, we were able to show a reduction in inflammatory markers upon co-treatment with A1AT in LPS- and Aβ-treated primary microglial cells. The strong immune reaction obtained with LPS in primary microglial cells could be reduced dramatically with A1AT. In our study, this suppression of immune reaction was shown by the analysis of the secreted amount of TNF-α, IL-6, IL-1β and NO.

The established, classic signaling pathways via the phosphorylation of the MAPKs p38, p44/42 or JNK were not involved here because A1AT co-treatment had no effect on the phosphorylation state of the aforementioned MAPKs. As A1AT has been shown to raise intracellular cAMP levels via PKA in monocytes [11], and PKA in turn plays also a role in inflammatory signaling [4,29], we investigated the effect of A1AT on cAMP/PKA/phospho-CREB signaling. Interestingly A1AT enhances the phosphorylation of CREB, which can be antagonized by the PKA inhibitor H89. However, this antagonism is not reflected in the cytokine levels. TNF-α-levels could not be restored with the PKA inhibitor H89. An involvement of the non-canonical cAMP signaling pathway via EPAC has also been excluded with specific inhibitors (data not shown).

The inhibition of intracellular calcium levels may well be the reason for the reduction in the pro-inflammatory mediators that we observed in our study [6,7]. Indeed, by using a fluorescent calcium indicator (Fluo-4), we were able to observe a reduction in intracellular calcium levels of BV-2 microglial cells upon administration of A1AT over time. Reduced levels of intracellular calcium might contribute to the reduction of pro-inflammatory mediators in our study, but further experiments are needed to prove this theory. However, as mentioned before, calpain is activated by calcium [17], and we also observed an inhibition of calpain activity upon treatment with A1AT. Thus, A1AT could inhibit calpain via a reduction of intracellular calcium levels.

Additional studies on calpain activity and inflammation demonstrated that IκB, the inhibitor of the transcription factor NF-κB, might be a substrate of calpain [19]. Therefore, high levels of activated calpain disinhibit NF-κB from its translocation to the nucleus and induce gene expression of several pro-inflammatory cytokines, including TNF-α and IL-6. However, further studies are necessary to elucidate whether this molecular mechanism also occurs in microglial cells.

After having seen impressive anti-inflammatory effects and elucidated the mechanisms of A1AT on microglial-mediated neuroinflammation, we further analyzed the effect of A1AT on the toxicity, oligomerization and phagocytosis of Aβ. We found a protective, concentration-dependent effect of A1AT on the viability of Aβ_1-42_ oligomer-treated primary microglial cells. Interestingly, the lowest concentration of 0.5 mg/ml A1AT, which is consistent with the serum A1AT concentration of A1AT deficiency patients [30], had no protective effect. To distinguish between an extracellular or intracellular effect, we checked the oligomerization state upon co-incubation with A1AT and a possible influence of A1AT on the oligomerization state after the oligomerization process, respectively. No change in the oligomerization state could be observed in any of these experimental conditions. This finding is in contrast to the study of Giunta *et al*., who could show a small effect of A1AT on the fibrillation of Aβ_25-35_ and a protective effect on the toxicity of Aβ_25-35_ in a human red blood cell lysis model [23]. The observed reduction in calpain activity upon A1AT treatment might account for the increased cell survival: enhanced calpain activity is known to induce cell death [31].

A key feature of microglial cells is their capability of phagocytosis, which supports brain homoeostasis by degrading cellular debris and also potentially neurotoxic substances. Impaired clearance of toxic Aβ by microglial cells can be observed in AD and is interpreted as one pathological hallmark of the disease [32]. Here, we could observe a decreased uptake of Aβ by microglial cells following incubation with A1AT. This result is in line with previous findings, that calpain activation is an important step to induce phagocytosis by monocytes [33]. Blocking calpain activation by A1AT, as shown in this study, should therefore result in a decreased capability of phagocytosis. Of course, this is an undesired effect and further experiments are needed to consider the positive effects of A1AT regarding cytokines and toxicity versus the negative effect on Aβ phagocytosis. An effect of A1AT on the expression of Aβ degrading enzymes in this experimental setting is not very likely due to the short incubation time of only three hours.

## Conclusions

This *in vitro* study of the effect of A1AT on microglial cells shows a protective and anti-inflammatory effect on microglial cells. Besides the positive effect on the viability of Aβ-treated cells, A1AT also reduces the release of pro-inflammatory mediators. We assume that both effects are mediated via inhibitory effects on intracellular calcium levels and calpain activation, yet the particular effectors upstream of this inhibition still have to be identified and are subject of further studies. The inhibition of Aβ-induced microglial cytotoxicity as well as the release of pro-inflammatory cytokines by A1AT can be rated as beneficial to slow down neurodegenerative processes in AD. However, we could also observe a decreased ability to take up neurotoxic Aβ by microglial cells following incubation with A1AT, which may be seen as an unsolicited effect regarding immune-modulatory effects of the substance. Further studies are needed to elucidate the effects of A1AT in neurodegenerative processes - especially in context with neuronal cells. These findings support the importance of the role of A1AT in Alzheimer-associated neuroinflammation and the potential of A1AT to transfer this knowledge for further investigation in transgenic AD animals.

## Abbreviations

A1AT: α_1_-antitrypsin
Aβ: Amyloid-β
AD: Alzheimer disease
CREB: cAMP response element binding protein
DMEM: Dulbecco’s modified Eagle’s medium
ELISA: Enzyme-linked immunosorbent assay
FBS: Fetal bovine serum
GAPDH: Glyceraldehyde 3-phosphate dehydrogenase
HBSS: Hank’s buffered salt solution
IL: Interleukin
JNK: c-Jun N-terminal kinase
LPS: Lipopolysaccharide
MAPK: Mitogen-activated protein kinase
MTT: 3-(4,5-dimethylthiazol-2-yl)-2,5-diphenyltetrazolium bromide
NFκB: Nuclear factor κB
NO: Nitric oxide
PKA: Protein kinase A
RFU: Relative fluorescence units
SAPK: Stress-activated protein kinase
TLR: Toll-like receptor
TNF-α: Tumor necrosis factor-α
